# MUC4 Overexpression Augments Cell Migration and Metastasis through EGFR Family Proteins in Triple Negative Breast Cancer Cells

**DOI:** 10.1371/journal.pone.0054455

**Published:** 2013-02-11

**Authors:** Partha Mukhopadhyay, Imayavaramban Lakshmanan, Moorthy P. Ponnusamy, Subhankar Chakraborty, Maneesh Jain, Priya Pai, Lynette M. Smith, Subodh M. Lele, Surinder K. Batra

**Affiliations:** 1 Department of Biochemistry and Molecular Biology, University of Nebraska Medical Center, Omaha, Nebraska, United States of America; 2 Department of Biostatistics, University of Nebraska Medical Center, Omaha, Nebraska, United States of America; 3 Department of Pathology and Microbiology, University of Nebraska Medical Center, Omaha, Nebraska, United States of America; 4 Eppley Institute for Research in Cancer and Allied Diseases University of Nebraska Medical Center, Omaha, Nebraska, United States of America; Roswell Park Cancer Institute, United States of America

## Abstract

**Introduction:**

Current studies indicate that triple negative breast cancer (TNBC), an aggressive breast cancer subtype, is associated with poor prognosis and an early pattern of metastasis. Emerging evidence suggests that MUC4 mucin is associated with metastasis of various cancers, including breast cancer. However, the functional role of MUC4 remains unclear in breast cancers, especially in TNBCs.

**Method:**

In the present study, we investigated the functional and mechanistic roles of MUC4 in potentiating pathogenic signals including EGFR family proteins to promote TNBC aggressiveness using *in vitro* and *in vivo* studies. Further, we studied the expression of MUC4 in invasive TNBC tissue and normal breast tissue by immunostaining.

**Results:**

MUC4 promotes proliferation, anchorage-dependent and-independent growth of TNBC cells, augments TNBC cell migratory and invasive potential *in vitro*, and enhances tumorigenicity and metastasis *in vivo*. In addition, our studies demonstrated that MUC4 up-regulates the EGFR family of proteins, and augments downstream Erk1/2, PKC-γ, and FAK mediated oncogenic signaling. Moreover, our studies also showed that knockdown of MUC4 in TNBC cells induced molecular changes suggestive of mesenchymal to epithelial transition. We also demonstrated in this study, for the first time, that knockdown of MUC4 was associated with reduced expression of EGFR and ErbB3 (EGFR family proteins) in TNBC cells, suggesting that MUC4 uses an alternative to ErbB2 mechanism to promote aggressiveness. We further demonstrate that MUC4 is differentially over-expressed in invasive TNBC tissues compared to normal breast tissue.

**Conclusions:**

MUC4 mucin expression is associated with TNBC pathobiology, and its knockdown reduced aggressiveness *in vitro,* and tumorigenesis and metastasis *in vivo*. Overall, our findings suggest that MUC4 mucin promotes invasive activities of TNBC cells by altering the expression of EGFR, ErbB2, and ErbB3 molecules and their downstream signaling.

## Introduction

Triple negative breast cancers (TNBCs) represent approximately 10–17% of all breast cancer incidents [1]. In comparison to the luminal subtypes, TNBCs are associated with poor prognosis [2], short survival, and high recurrence rates after adjuvant therapy [3]. TNBCs are associated with increased risk for visceral [4] and brain metastases, and also require more aggressive treatment. Although several therapeutic options targeting EGFR, PARP1, VEGF-α, Src, HDAC, and MEK are being investigated in clinical trials [5], the overall prognosis of patients with TNBC remains dismal owing to a lack of effective treatment. Thus, there is an urgent need to investigate the underlying molecular mechanisms responsible for the aggressive nature of TNBC, and to develop targeted approaches for treatment of invasive TNBCs.

Epithelial cells produce mucins to lubricate and protect themselves from extrinsic physical and biological assaults [6]. However, aberrant expression of mucins has been reported to promote cancer development, and affects cellular growth, transformation, and invasion [7]. Aberrantly over-expressed membrane-tethered mucins, including MUC1 [8] and MUC4 [6], play diverse functional roles in several epithelial cancers, including ovarian, pancreatic [9], [10], and breast [11]. We have previously demonstrated that MUC4 enhances tumorigenicity and metastasis in pancreatic [7], [12] and ovarian [10] cancer. Furthermore our studies have established that MUC4 is associated with drug resistance in pancreatic cancer [13], [14]. An earlier study reported that there is a high incidence of MUC4 expression in breast cancer [15], which is associated with metastatic disease [16]. However, inadequate information is available regarding the functional role(s) of MUC4 mucin in breast cancer especially in TNBC.

In the current study, we investigated the role of MUC4 in TNBC by *in vitro* and *in vivo* functional studies, and by studying the expression of MUC4 in TNBC tissue. MUC4 potentiated oncogenic signals to promote proliferation, growth, motility, and invasiveness of TNBC cells *in vitro*, and tumorigenicity and metastasis *in vivo*. Furthermore, compared to normal breast tissues, MUC4 expression was overexpressed in invasive TNBC tissues. Thus, we propose that MUC4 mucin is a new potential target for treatment of invasive and metastatic TNBCs.

## Materials and Methods

### Cell Culture

MDA-MB-231 (HTB-26), BT-20 (HTB-19), and MDA-MB-468 (HTB-132) parental cell lines were a generous gift from Dr. Vimla Band, University of Nebraska Medical Center, Omaha, NE, USA. These cell lines are commercially available in American Type Culture Collection (ATCC) and already published in many research articles. A limited number of passages of ATCC characterized cells was maintained, tested to be free of mycoplasma contamination, and cryopreserved. All experiments were performed with cells at less than 20 passages after receipt. The α-MEM media (Invitrogen, Carlsbad, CA) supplemented with 10% fetal bovine serum, 100 U/ml penicillin, 100 µg/ml streptomycin, 2 mM L-glutamine, 10 mM HEPES (pH 7.4), and 1× NEAA was used for maintaining MDA-MB-231 cells and also to maintain sustained MUC4 expression. After stable transfection and clone selection, control MDA-MB-231-SCR and MUC4 knockdown MDA-MB-231-shMUC4 cells were maintained in complete α-MEM media with the addition of 1–3 µg/ml puromycin.

### Stable Silencing of MUC4 using a Plasmid Construct (pSUPER-retro-puro-shMUC4)

The method of generating the shRNA construct for *in vitro* stable knockdown of MUC4 has been described previously [7]. Briefly, phoenix packaging cells were transfected with the pSUPER-retro-puro vector containing either the MUC4 shRNA insert (pSUPER-retro-puro-shMUC4) or a scrambled sequence (pSUPER-retro-puro-SCR) using FuGENE 6 (Invitrogen) following the manufacturer's protocol. Media containing infection-competent retroviruses containing supernatant were collected 48 h after transfection. Polybrene (4 µg/mL) was added with the retroviruses to enhance the target cell infection efficiency. Cells (MDA-MB-231) were plated in 100 mm dishes at 60% confluence and infected with the retroviruses. Stable pooled populations of MDA-MB-231-SCR (control) and MDA-MB-231-shMUC4 (MUC4 knockdown) cells were generated by selection using puromycin, and levels of mRNA transcripts, expression of protein, and the phenotype of cells were analyzed. The control and MUC4 knockdown cells were used for all functional studies.

### Growth Kinetic Studies

Growth kinetics and population doubling time of control and MUC4 knockdown cells were determined as described previously [12]. Briefly, for growth curves, control and MUC4 knockdown cells were seeded at 1×10^4^ cells/well in 6-well-plate in triplicate. Viable cells of control and MUC4 knockdown populations in each well of the 6-well plates were counted for 7 days by a viable cell counter (ViCell Coulter counter, Beckman Coulter, Inc., Brea, CA). Population doubling times of control and MUC4 knockdown cells were calculated from the number of cells growing in the log phase (96–144 h) and using the formula: T_d_ = 0.693t/ln (N_t_/N_0_), where t is time (in h), N_t_ is the cell number at time t, and N_0_ is the cell number at initial time.

### Colony Forming Assay

Colony forming assays were performed as described previously [17]. Briefly, colony-forming efficiency was examined 14 days after plating 250 cells/60 mm dish in quadruplicate, by staining with crystal violet (Sigma, St. Louis, MO). Colonies of >50 µm in size were counted using quantity One software (Bio-Rad, Richmond, CA, USA). Results are an average of 3 independent experiments.

### Assay for Anchorage Independent Growth in Soft Agar

Anchorage-independent growth assays were performed as described previously [18]. Briefly, 2.5 × 10^4^ cells of control and MUC4 knockdown cells were plated in 6-well plates in 1.5 mL of 0.35% low melting agarose (Sigma) in α–MEM media on top of a bottom layer of 0.5% agarose in α–MEM media. Plates were incubated for 2 weeks. Phase-contrast images were obtained under 40× magnification, and colonies were counted and plotted. Control and MUC4 knockdown cells were used for each experiment in triplicate. At least two independent experiments were performed.

### Immunoblot Assays

Protein extraction and immunoblotting were performed using standard procedures with control and MUC4 knockdown cells for EGFR, ErbB2, ErbB3, ErbB4, β–catenin, cyclin D1, CK-18, vimentin, vitronectin, ERK, FAK, and β-actin expression. 2% SDS-agarose gel electrophoresis was performed for MUC4 using 25 µg protein samples under reducing conditions as described previously [19]. ErbB2 (sc-52349), ErbB3 (sc-7390), ErbB4 (sc-8050), cyclin D1(sc-718), vitronectin (sc-28929), FAK (sc-557), p-FAK (sc-7383), HSC70 (sc-7298), Sprouty 2 (sc-30049) were purchased from Santa Cruz Biotechnology, CA, US. Anti-CK-18 (K0199-21) was purchased from US Biological, MA, US); antibodies against vimentin (V-2258), β-actin (A-2066) were purchased from Sigma-aldrich, St. Louis, MO, US; Erk1/2 (9194); anti-p-Erk1/2 (9101) was purchased from Cell signaling, MA, US; anti-PKCγ (ab71558) was from Abcam, Cambridge, MA, US; anti-Zonula occludens-1 (40–2300) from Invitrogen, Carlsbad, CA. EGFR (ICI). Anti-MUC4 antibodies 8G7 and 2214 were generated and characterized in our laboratory [20]. β-catenin antibody was from kind gift Dr. Keith Johnson, UNMC, NE.

### Three-dimensional Morphogenesis Assay and Confocal Imaging

The three-dimensional Matrigel assays were performed following the method described previously [21]. Approximately, 2.5 × 10^3^ cells per well, as single cell suspensions, were plated onto an eight-well chamber slides on top of a polymerized layer of 100% growth factor reduced Matrigel (BD BioSciences, San Jose, CA, US), with 0.4 mL assay media containing 2% Matrigel using the overlay method [22]. Stable transfactants were cultured with puromycin. The medium was changed after every 3 days for each set. 3D structures formed by both control and MUC4 knockdown cells were quantified. Acinar-like structures were defined as regular, round structures that were clearly identified by bright field microscopy at 20× magnification; protrusive structures were defined as those exhibiting one or more multicellular outgrowths clearly invading the surrounding Matrigel. This observation was further confirmed by staining with junctional protein zonula occludens-1 (ZO-1). Immunofluorescence microscopy was performed following a standard methodology as described previously [21].

### Quantitative Real-time Polymerase Chain Reaction

Quantitative real-time PCR was performed using standard procedures using a LightCycler 480 SYBR Green 1 Master (Roche, Germany) with specific primers as described previously [23]. The forward and reverse primers for MUC4, and β-actin were custom synthesized from IDT technology (Integrated DNA Technology, Coralville, IA, USA) and are listed in Table S2.

### Immunohistochemistry

Immunohistochemical analysis of tumor microarrays and sections, obtained from tumors generated by orthotopic implantation of control and MUC4 knockdown cells, was performed as described previously [24]. Briefly, after baking at 56°C overnight, the tissues were dewaxed in xylene twice for 5 min, followed by rehydration through graded ethanol. Endogenous peroxidase activity was quenched by incubating the slides with 3% hydrogen peroxide in methanol for 30 min. Antigen retrieval was performed in 0.01 M pre-heated citrate buffer (pH 6.0, 95°C) in a microwave for 15 min. Non-specific reactivity with the antibody was blocked by incubating the slides with horse serum (ImmPRESS Universal Antibody Kit, Vector Labs, Burlingame, CA, USA) for 2 h followed by the addition of the primary anti-MUC4 antibody [1∶200] (Mouse monoclonal antibody 2214; 1.66 mg/ml). After overnight incubation at 4°C, the slides were washed with PBS and incubated with universal secondary antibody (ImmPRESS Universal Antibody Kit, Vector Labs) for 30 min. Staining was visualized by adding 3, 3′-diaminobenzidine solution (DAB Substrate Kit, Vector Labs). The slides were counterstained with Gill’s hematoxylin (Vector Labs) and dehydrated in graded ethanol and washed with xylene. The slides were mounted with a few drops of permanent mounting medium (Permount, Fisher Scientific, Pittsburgh, PA, USA). All slides were observed under a Nikon light microscope and photographs of representative areas taken with the Q-capture Micropublisher 5.0 camera (Leeds Precision Instruments, Minneapolis, MN, USA) using the Q-capture suite software package (QImaging, Surrey, BC, Canada). The intensity of MUC4 expression was graded on a scale of 0 to 3 (0: no staining; 1+: weakly positive; 2+: moderately positive; 3+: strongly positive). The percentage of MUC4-positive cells was quantified.

### Immunofluorescence Staining and Confocal Laser Scanning Microscopy

Confocal analysis of control and MUC4 knockdown cells was performed as described previously [25]. Briefly, cells were grown onto 18 mm glass coverslips aseptically and fixed with 3.7% paraformaldehyde and permeabilized with 0.5% Triton X-100. Methanol (100%, ice chilled) was used for MUC4 staining. Next, 10% goat serum for 1 h in PBS was added to block nonspecific binding sites. The primary antibody anti-MUC4 (1.86 µg/mL, 8G7; an antibody generated in our laboratory) was added to coverslips at 1∶200, and then incubated overnight at 4°C in 1% goat serum in PBS. Alexa fluor-conjugated secondary antibody (Jackson ImmunoResearch, West Grove, PA, USA) was added at 1∶1000 in 1% goat serum in PBS for 1 h. DAPI staining was done during mounting. After washing in PBS and then water, images were captured and analyzed with a laser scanning confocal microscopy (Zeiss LSM 510 META, Carl Zeiss Microscopy GmbH, 07740 Jena, Germany).

For F-actin (filamentous actin) staining, phalloidin (fluorescent phallotoxins from Invitrogen) was used as described in the manufacturer’s protocol. Briefly, cells were grown onto sterile 18 mm glass coverslips and fixed with 3.7% formaldehyde and permeabilized with 0.5% Triton X-100. Phalloidin at 1∶40 in PBS was used to stain F-actin for 1 h. DAPI staining was done during mounting. Images were captured and analyzed with a laser scanning confocal microscopy (Zeiss LSM 510 META).

### Cell Cycle Analysis

Cell cycle analysis was performed by flow cytometry using standard procedures as described previously [17]. Briefly, cells were serum starved for 48 hours and re-stimulated with serum for 24 hours. After serum re-stimulation, cells were harvested with a PBS-based enzyme-free cell dissociation buffer (Invitrogen) followed by HPBS washing. MDA-MB-231-SCR and MDA-MB-231-shMUC4 cells were fixed for 1 hour in 70% ethanol, washed 3× with HPBS (pH 7.4), and resuspended in Telford reagent composed of propidium iodide (50 µg/ml) supplemented with EDTA (90 mM), Triton X-100 (0.1%), and RNase A (1 µg/ml). DNA content was measured using a FACScan cytometer (FACStar, Becton Dickinson, Franklin Lakes, NJ, USA). All samples were analyzed in triplicate, and the data presented are the average of the three independent experiments.

### Wound Healing and Trans-well Migration Assay

The motility assay was performed as described previously [26]. Briefly, control and MUC4 knockdown cells (1×10^5^/well) were seeded in 12-well plates. Cells were incubated in serum-free medium for 32–48 h prior to generating the wound by scraping with a plastic tip across the cell monolayer. Cells were incubated for 12 h and phase contrast images were recorded in ten different fields at the time of wounding (0 h) and 12 h thereafter. After 12 h, the migration of control and MUC4 knockdown cells was measured. The results presented are the average of two independent experiments. Trans-well migration assays were performed as described previously [27] using a chamber with monolayer-coated polyethylene terephthalate membranes (24-well insert, pore size of 8 µm) for both control and MUC4 knockdown cells. The results presented are the average of three experiments.

### Cell Invasion Assay

The invasion assay was performed as described previously [28] using a chamber with Matrigel-coated membrane inserts (24-well insert, pore size of 8 µm) for both control and MUC4 knockdown cells.

### Microarray Gene Expression Analysis

Total RNA was isolated using RNeasy Mini Kit columns as described by the manufacturer (Qiagen, Valencia, CA, US). RNA yield and purity were measured photo-metrically using nanodrop (NanoDrop 1000 spectrophotometer, Thermo Scientific, Wilmington, Delaware USA) and also in criterion gel (Bio-Rad). Spotted microarrays were used to identify differentially expressed genes between MDA-MB-231-SCR (control) and MUC4 knockdown MDA-MB-231-shMUC4 samples. After reverse transcription with SuperScript II, cDNA was transcribed and control and MUC4 knockdown samples were labeled with Cy3 and Cy5 respectively, and hybridized to HOA_005_0001 human OneArray DNA microarrays (Phalanx Biotech, CA, USA) containing 30,275 features probing for approximately 22,000 unique genes, according to standard procedures followed at the Microarray Core Facility of the University of Nebraska Medical Center (UNMC), Omaha, NE. A universal human reference (Stratagene, Cat: 740000, Cedar Creek, TX, US) was used for normalization. Microarrays were scanned with the GenePix 4000B Scanner (Axon Instruments, Foster City, CA, US). The Gene Expression Omnibus (GEO) accession number for our micro array data is **GSE40157.** (http://www.ncbi.nlm.nih.gov/geo/query/acc.cgi?token=nfqldmkagcqwcru&acc=GSE40157).

### Data Analysis and Real Time Validation

Data analysis and real time validation were performed using standard procedure as described previously [23]. Briefly, array quality control, statistical data analysis, and data visualization were performed at the University of Nebraska Medical Center using standard settings. Spot filters, background subtraction, and lowess normalization were applied prior to data analysis through BRB ArrayTools developed by Dr. Richard Simon and Amy Peng [29]. Genes were excluded if any of the spots were missing for any of the samples. Random-variance paired t-tests were used to determine those genes that were differentially expressed between control and MUC4 knockdown samples, by comparing the log Cy3 (control) and Cy5 (MUC4 knockdown) channel intensities. A significance level of 0.001 was selected to limit the false discovery rate due to multiple comparisons.

The microarray results were validated by RT-PCR. All RT-PCR reactions were performed using SYBR green based chemistry. For validation, eight of the differentially expressed genes, 4 up-regulated (COL4A5, SMAD6, CXCL1, and DUSP2) and 4 down-regulated (A100A4, PDGFRB, SOCS2, and PLCXD1), detected by microarray were selected. Validation was done using randomly selected original samples (submitted for microarray analysis) and in freshly isolated RNA from both control and MUC4 knockdown cells. Relative gene expression was determined using the 2-δδCT method. Primers were custom synthesized from IDT technology and are listed in Table S2.

### *In vivo* Tumorigenesis and Metastasis in Nude Mice

To test tumorigenicity and metastasis, control and MUC4 knockdown cells (0.1 × 10^6^) were orthotopically injected into the mammary fat pads of nude female mice (The Jackson Laboratory, Bar Harbor, ME, USA; and *n* = 9 for each group) and the growth of tumors was followed for 8 weeks using procedures described previously [30]. After 8 weeks, mice were euthanized according to IACUC (Institutional Animal Care and Use Committee) guidelines and checked for tumor size and metastasis. To confirm reduced metastasis by MUC4 knockdown cells, 0.3 × 10^6^ cells were orthotopically injected into the mammary fat pads of nude female mice (*n* = 6). After 8 weeks, animals were euthanized as above and checked for tumor size and metastasis. The results presented are the average of two independent experiments. Institutional Animal Care and Use Committee at University of Nebraska Medical Center has approved to proceed the above mentioned experiments. The approval number is 12-031-FC.

### Statistical Analysis

Data were analyzed using two-tailed Student's *t*-tests and two-tailed Fisher's exact tests where appropriate in Microsoft Excel 2010. The software used for the Wilcoxon rank sum test is SAS software (SAS Institute Inc., Cary, NC). P<0.05 was considered statistically significant.

## Results

### MUC4 Promotes Proliferation and Growth

**Figure 1:**
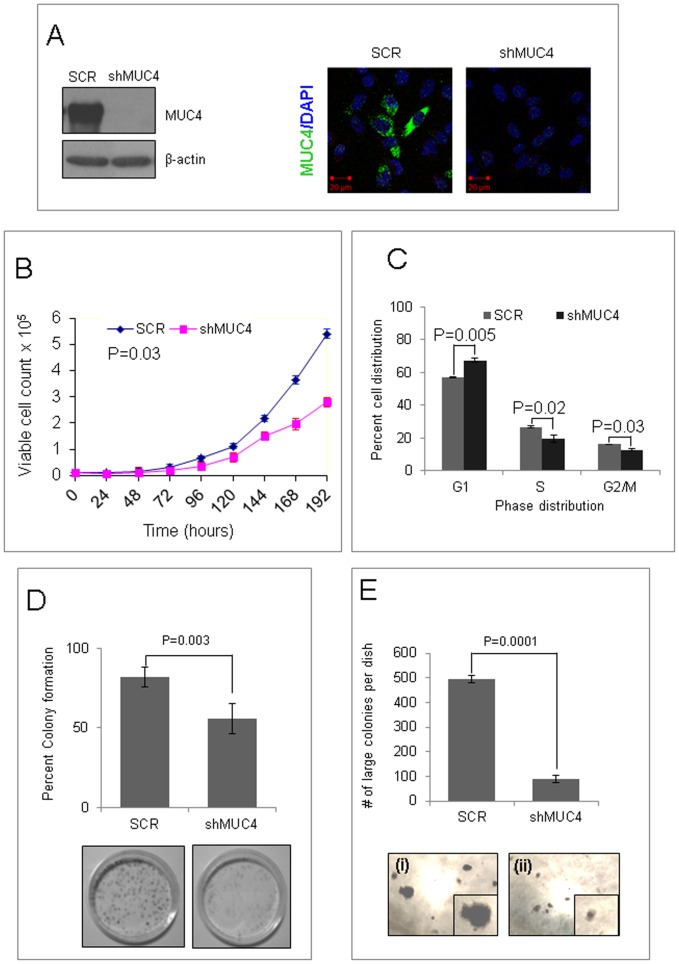
MUC4 promotes proliferation and growth of MDA-MB-231 cells. (**A**) Detection of MUC4 protein expression in control (MDA-MB-231-SCR) and MUC4 knockdown (MDA-MB-231-shMUC4) cells. Immunoblot showed reduced expression of MUC4 in MDA-MB-231-shMUC4 cells compared to control cells. Immunostained cells using human anti-MUC4 mouse monoclonal antibody (8G7) showed reduced expression of MUC4 in MDA-MB-231-shMUC4 cells compared to control cells. (**B**) In proliferation analyses, when the number of cells was plotted against the incubation period (hours), control cells showed a significantly higher proliferation rate than MUC4 knockdown cells, p = 0.03. Population doubling time of control cells was less than MUC4 knockdown cells when calculated from the number of cells growing in log phase (day 2 to 6) using the formula, T_d_ = 0.693t/ln (N_t_/N_0_). (**C**) Cells, following synchronization and serum re-stimulation and stained with Telford reagent (containing propidium iodide) and analyzed by FACS, showed that the number of MUC4 knockdown cells in the G1 phase was higher than control cells suggesting the inhibition of cell cycle progression. (**D**) The colony forming ability of control cells was higher than MUC4 knockdown cells under anchorage-dependent conditions. After staining, colonies of >50 µm in size were counted using Quantity One software, p = 0.003. Images of anchorage–dependent growth assays were shown at the bottom. (**E**) The colony forming ability of control cells was higher than MUC4 knockdown cells under anchorage-independent conditions. Colonies were counted and plotted. Columns: mean of triplicates; bars: SD, p = 0.0001. Phase-contrast images were recorded at 10× magnification. Microscopic images of colonies found in anchorage–independent growth assays. Higher magnification of a typical colony was shown in box on bottom right corner to emphasize a (i) big colony with migratory outer cells versus (ii) smaller and compact colonies. SCR are control and shMUC4 are MUC4 knockdown cells. All data presented are the average of 3 independent experiments.

In a preliminary screening of breast cancer cells, we observed that the invasive MDA-MB-231 TNBC cell line expressed MUC4 when grown in α-MEM media (**Figure** 1A, left lane), while non-invasive TNBC cell lines BT-20 and MDA-MB-468 [31] were MUC4 negative (data not shown). Thus, we chose MDA-MB-231 cells for further studies and generated a line with stable knockdown of MUC4 using a retroviral construct [7] to elucidate the functional significance of MUC4 in TNBC pathophysiology. A stable line generated with non-targeted scrambled shRNA (SCR) was used as control for all experiments performed in this study. Knockdown of MUC4 was confirmed by real-time PCR (data not shown), immunoblotting, and immunofluorescence analyses (**Figure** 1A). Immunoblot analysis indicated a ∼95% down-regulation of MUC4 at the protein level in MUC4 knockdown cells (MDA-MB-231-shMUC4) as compared with control cells (MDA-MB-231-SCR) cells.

Proliferation is an important cellular event in cancer cells. Growth kinetic analysis showed that MUC4 knockdown cells had a significantly lower (p = 0.03) proliferation rate with a doubling time of 44 hours compared with control cells, which had a doubling time of 32 hours (**Figure** 1B). Proliferation in cancer cells is mostly driven by alterations in the cell cycle or apoptosis or both [32]. Therefore, we investigated the effect of MUC4 down-regulation on cell cycle progression and apoptosis. Cell-cycle analyses revealed that 57% of control cells were in the G1 phase, with 16% of cells in the G2/M phase (**Figure** 1C, please see **Figure** S1A). In contrast, 67% of MUC4 knockdown cells were in G1, with 12% in G2M (p = 0.005 and p = 0.037, respectively). Thus, knockdown of MUC4 resulted in the accumulation of cells in the G1 phase and inhibited their transition to G2/M via the S phase. However, no significant change in apoptosis was observed following MUC4 knockdown (**Figure** S1B). Under anchorage-dependent conditions, MUC4 knockdown cells exhibited significantly lower (p = 0.003) colony-forming ability (56%) compared with control cells (82%) (**Figure** 1D). When analyzed for anchorage-independent growth in soft agar, control cells formed numerous colonies (495±9 per plate) after 2 weeks. In contrast, MUC4 knockdown cells formed significantly fewer (90±8 per plate, p = 0.001) and smaller colonies, indicating that MUC4 expression contributes to a transformed phenotype of MDA-MB-231 cells (**Figure** 1E).

### MUC4 Up-regulates the EGFR Family of Proteins and Induces Downstream Signaling

**Figure 2:**
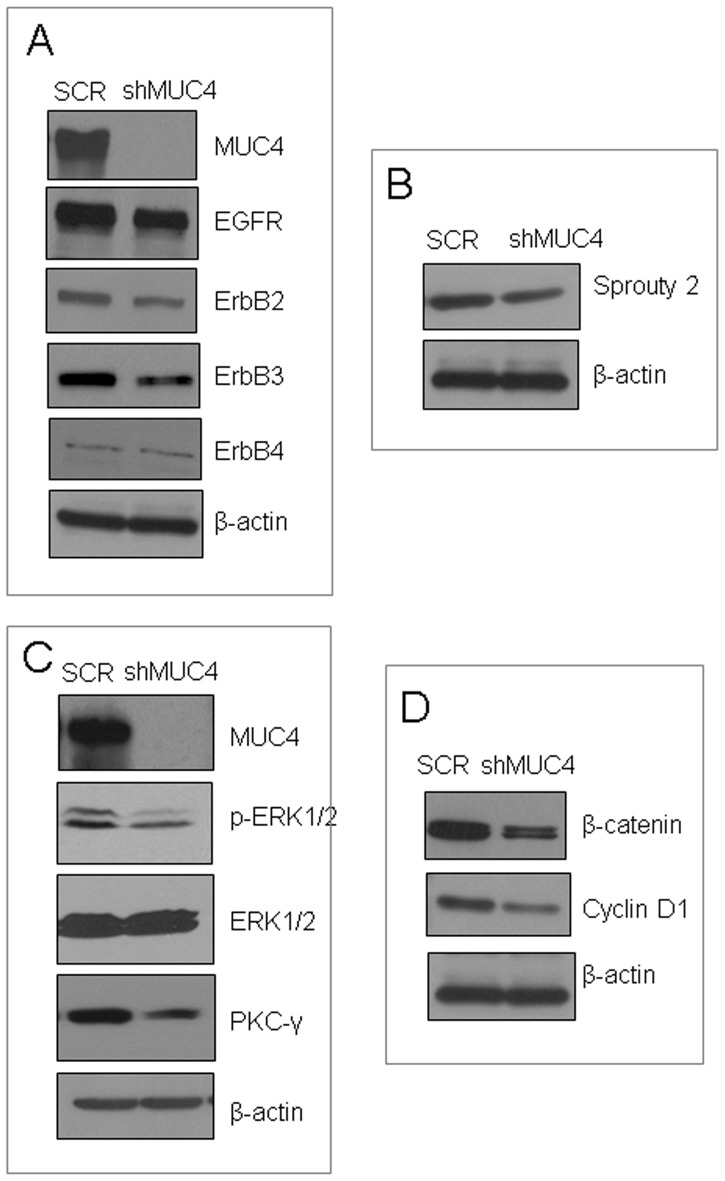
MUC4 up-regulates EGFR family receptors and induces downstream Erk1/2 and PKC-γ pathways. (**A**) Immunoblot analyses showed reduced expression of EGFR, ErbB2, and ErbB3 in MUC4 knockdown cells compared with control cells. (**B**) Reduced expression (using immunoblot) of Sprouty 2 was detected in MUC4 knockdown cell when compared with control cells. (**C**) Immunoblot showed that reduced phosphorylation of Erk1/2 and expression of PKC-γ in MUC4 knockdown cells compared with control cells. β-actin was used as a loading control. (**D**) Immunoblot analyses showed reduced expression of β-catenin and its target gene product cyclin D1in MUC4 knockdown compared with control cells.

EGFR plays important roles in the proliferation of TNBC [33], while MUC4 has been demonstrated to stabilize another EGFR family member, ErbB2 [25]. Thus, we studied the effect of MUC4 knockdown on the status of EGFR family members and downstream signaling. Knockdown of MUC4 resulted in reduced expression of ErbB1 (EGFR) and ErbB3, whereas ErbB4 levels remained unchanged (**Figure** 2A). This result suggests that MUC4 may use an alternative mechanism to promote aggressiveness and metastasis of TNBC cells, because ErbB2 was present at low levels in TNBC cells. Sprouty 2 enhances EGFR stability by sequestering Cbl (Casitas B-lineage Lymphoma-an E3 ubiquitin ligase), and thus inhibiting ubiquitin-mediated degradation of EGFR [34]. We observed a decreased expression of Sprouty 2 in MUC4 knockdown cells compared with control cells (**Figure** 2B). Alteration of EGFR expression by MUC4 resulted in enhanced downstream signaling *via* Erk1/2 and PKC-γ pathways, as indicated by increased phosphorylation of Erk1/2 and increased expression of PKC-γ in control cells (**Figure** 2C). Furthermore, MUC4 knockdown resulted in decreased expression of cyclin D1 and its upstream regulator β-catenin, suggesting that MUC4 augments cell cycle progression possibly *via* cyclin D1(**Figure** 2D).

### MUC4 Enhances Migratory and Invasive Potential

**Figure 3:**
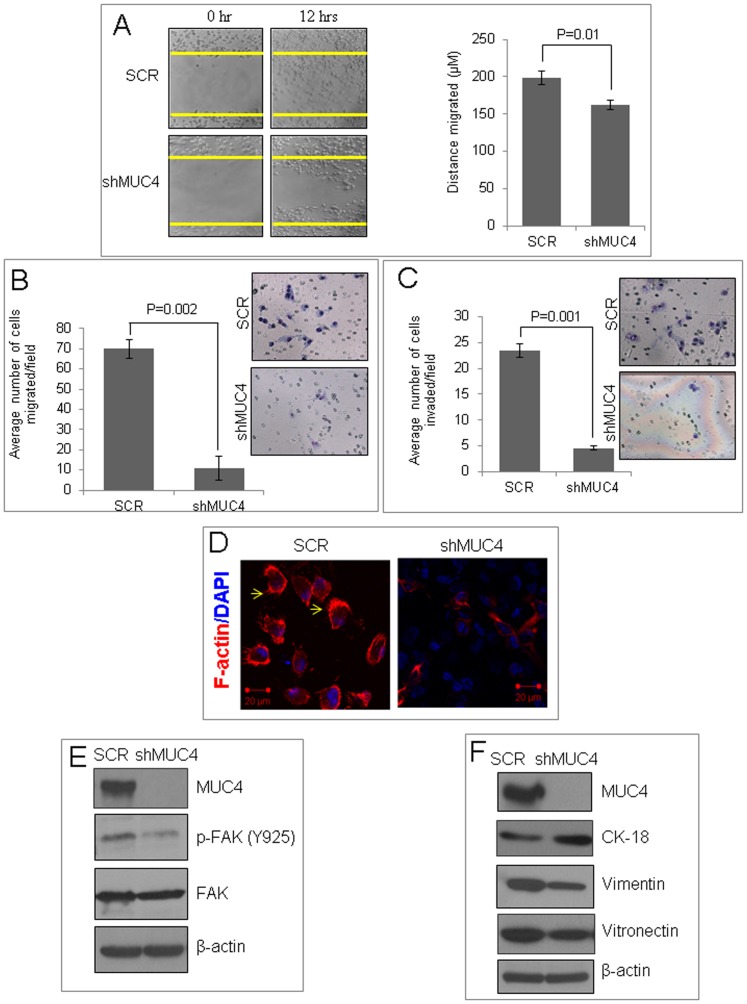
MUC4 enhances migratory and invasive potential. (**A**) After 24 hours serum starvation, a wound was created with a plastic tip on plates containing control or MUC4 knockdown cells. Cells were incubated in complete media for 12 hours. Motility of cells was photographed under bright-field microscopy (left, 10× magnification). After 12 hours, the migration of control cells and MUC4 knockdown cells was measured (in µm^2^) using DatInf Measure setup wizard software (http://tucows.texasonline.net). Values were calculated and plotted (right). (**B and C**) Control and MUC4 knockdown cells were serum starved for 48 h and seeded on non-coated or Matrigel-coated membranes for motility (**B**) and invasion (**C**) assays, respectively, and incubated for 24 h. Medium containing 10% fetal bovine serum in the lower chamber was used as a chemo-attractant. Cells that did not migrate through the Matrigel and/or pores in the membrane were removed using a cotton swab, and cells on the other side of the membrane were stained and photographed under bright-field microscopy (10× magnification). The number of cells that migrated and invaded was higher in control than the MUC4 knockdown cells. Data presented are the average number of cells/field for 10 fields. Columns: average of three independent experiments; bars: SE, p = 0.002 and p = 0.001, respectively. Representative images of control and MUC4 knockdown cells were shon in both figures. (**D**) Phalloidin staining showed that visualized F-actin under a laser scanning microscope is reduced in MDA-MB-231-shMUC4 cells compared with the control cells. (**E**) Immunoblot analysis showed reduced phosphorylation of FAK in MUC4 knockdown cells compared with the control cells. (**F**) Immunoblot analysis showed reduced expression of mesenchymal markers such as vimentin and vitronectin; and increased expression of CK-18 in MUC4 knockdown cells compared to the control.

In addition to enhanced proliferation, the aggressiveness of a malignant cell is determined by its migratory and invasive potential. MUC4 knockdown cells exhibited significant decrease in motility, trans-well migration, and invasion (p = 0.01, p = 0.002, and p = 0.001, respectively). The motility of cells, determined by their migration in the wound gap after 12 h, in the wound healing assay decreased by 18% following MUC4 knockdown (**Figure** 3A). Similarly, trans-well migration and Matrigel invasion (**Figure** 3B-C) of MUC4 knockdown cells was decreased by 58% and 65%, respectively. Since actin plays an important role in defining cell shape and orchestrating events related to cellular motility, we investigated the effect of MUC4 on actin cytoskeleton reorganization. Following cell staining with rhodamine-conjugated phalloidin, control cells exhibited more lamellipodial structures compared to MUC4 knockdown cells, which had reduced F-actin (**Figure** 3D) and decreased levels of phosphorylated (Y925) focal adhesion kinase (**Figure** 3E). These results strongly suggest that MUC4 facilitates the migratory and invasive potential of MDA-MB-231 cells by inducing the reorganization of actin filaments. Since alterations in cell motility and cytoskeleton reorganization are associated with epithelial-to-mesenchymal transition (EMT), we investigated whether MUC4 regulates EMT in MDA-MB-231 cells. MUC4 Knockdown resulted in increased expression of the epithelial marker CK-18, and decreased expression of mesenchymal markers vimentin and vitronectin (**Figure** 3F).

### MUC4 Contributes to an Altered Morphology of Colonies

**Figure 4:**
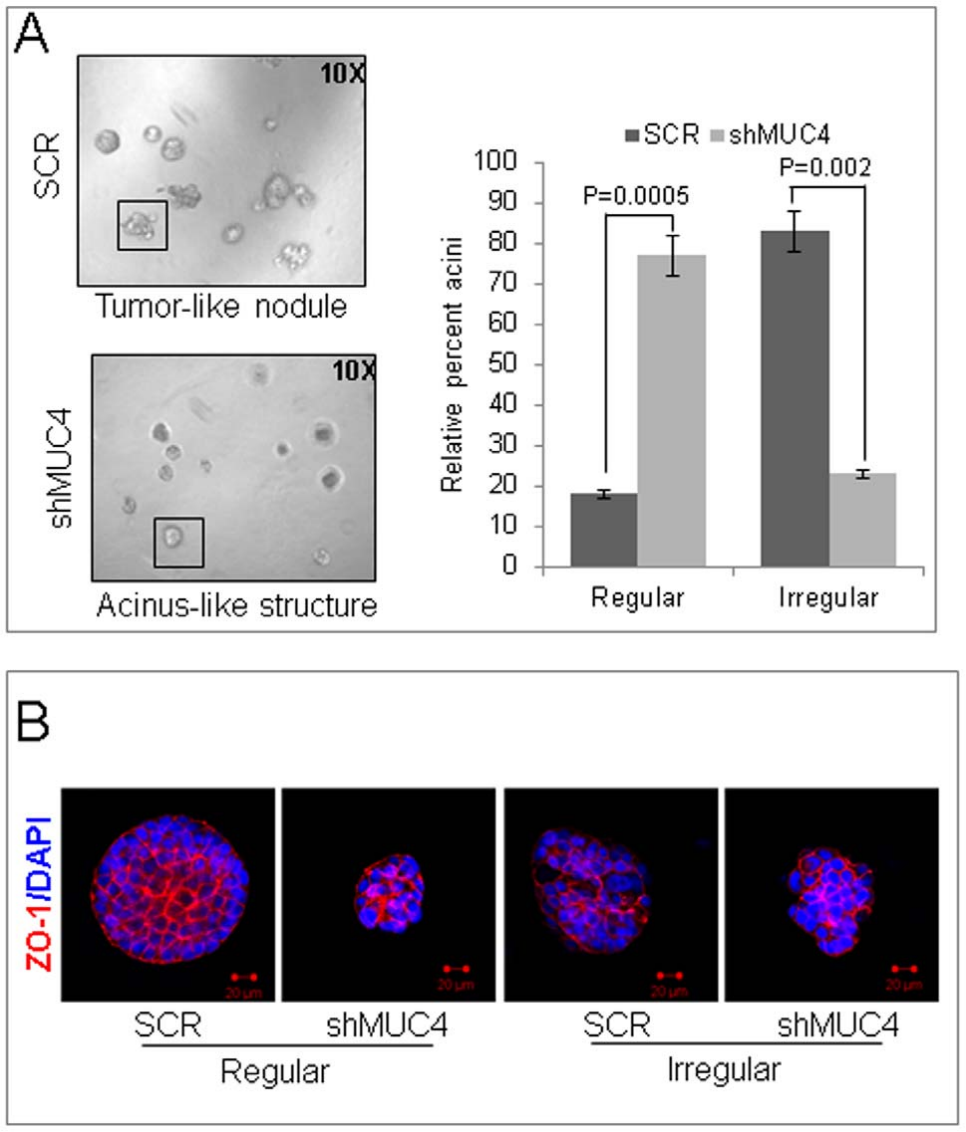
MUC4 contributes to a altered phenotype. (**A**) The control or MUC4 knockdown cells were seeded in 2% Matrigel on top of a 100% Matrigel layer, and fed with media every 3 days. After 7 days, acini-like structures were photographed under a phase-contrast microscope. The acini-like structures (examples shown in the boxes) that were regular (smooth and spherical shape) or irregular (irregular outline, multi-lobular) were counted and plotted as a percentage of the total count (p = 0.0005 for regular and p = 0.002 for irregular). A minimum of 120 structures was counted for each of control cells or MUC4 knockdown cells. Reduced irregular outline, multi-lobular and increased smooth and spherical shape colonies were found in MUC4 knock down cells when compared with control cells. (**B**) Structures were stained with the anti-ZO-1 antibody. 4,6-diamidino-2-phenylindole (DAPI) was used for nuclei staining. Optical sections (0.7–0.9 µm) were captured using a laser scanning confocal microscope. The images presented here are the central planes of the acini. Bar = 20 µm.

Epithelial cells in the mammary gland maintain a polarized morphology, specialized cell-cell contacts, and attachment to the underlying basement membrane. The development and maintenance of this polarized structure are critical for the formation and function of mammary epithelial cells [35]. However, the pathogenesis of tumors originating from epithelial cells requires the disruption of this intact and well-organized structural design. We used a 3D Matrigel culture model [36] to determine the effect of MUC4 knockdown on the morphology of the resulting 3D structures. The control cells failed to polarize in Matrigel and formed large, disorganized colonies. MUC4 knockdown did not induce structural polarization, but resulted in the formation of more organized structures reminiscent of mammary gland acini (**Figure** 4A). Confocal imaging of 3D Matrigel structures for ZO-1, a tight junction protein, further confirmed that control cells predominantly form disorganized and larger 3D colonies in Matrigel (83%) compared with the MUC4 knockdown cells (23%; p = 0.002) (**Figure** 4B). These results indicate that MUC4 induces the transformation of MDA-MB-231 cells to a highly migratory phenotype, and that stable MUC4 knockdown partially reduces this phenomenon.

### MUC4 Promotes Tumorigenesis and Metastasis

**Figure 5:**
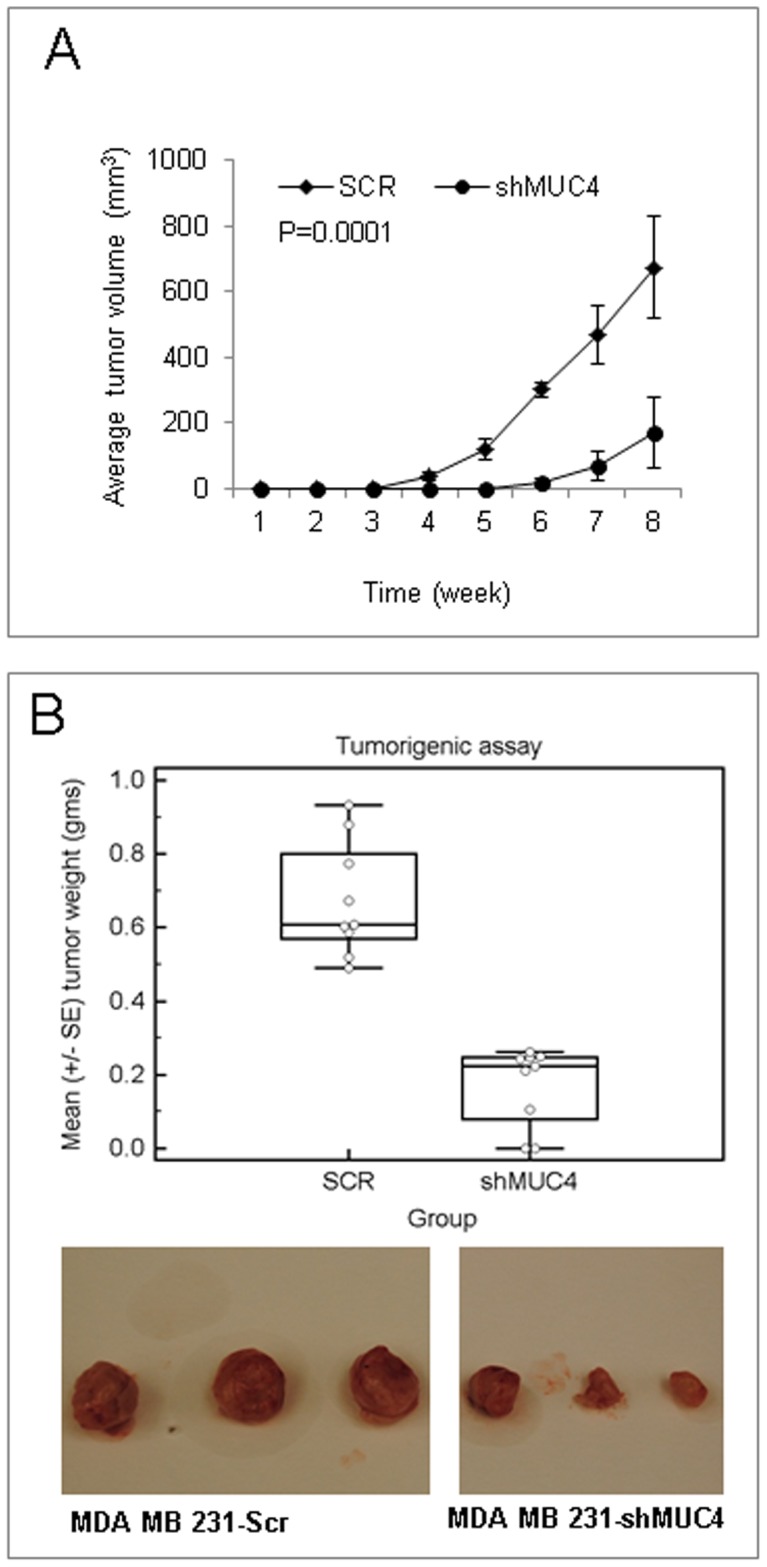
MUC4 promotes growth of MDA-MB-231 xenografts. (**A**) MUC4 knockdown and control cells (0.1 × 10^6^ cells/animal) were orthotopically implanted in mouse mammary fat pad of each mouse (right 3^rd^ mammary gland). Tumor volumes were calculated every week. The MUC4 knockdown cells started to grow tumors during the 5^th^ week, but control cells started to grow tumors during the 3^rd^ week, p = 0.0001. (**B**) Eight weeks after implantation, mice were sacrificed and tumors were excised and weighed. Stable silencing of MUC4 was found to decrease tumor growth, p = 0.0001.

As MUC4 knockdown was observed to augment proliferation, growth, migration, and invasion of MDA-MB-231 cells, we sought to investigate the effect of MUC4 knockdown on the tumorigenic and metastatic potential of MDA-MB-231 cells. Control and MUC4 knockdown cells were implanted orthotopically into mammary fat pads of two groups of female nude mice (n = 9). Control cells produced detectable tumors at week 3, while tumors resulting from MUC4 knockdown cells were detectable only after 5 weeks (**Figure** 5A). The tumor volume from MUC4 knockdown cells was significantly smaller (p = 0.0001) and the tumors excised at 8 weeks had markedly reduced weight, compared with tumors obtained from control cells (mean 0.171±0.05 g in MUC4 knockdown vs. 0.653±0.07 g in control cells) (**Figure** 5B). In addition, *in vivo* transgene expression in control cells was confirmed in excised tumors at the mRNA and protein levels (**Figure** S2A-B). Next, we determined the frequency of metastases in mice implanted with control or MUC4 knockdown cells. All mice, implanted with control cells, developed metastases to one or multiple sites. Metastasis was observed in 2 of nine mice of each organ such as lung, ovary, and peritoneum and; in 3 of nine of each site like mesenteric lymph nodes, and intestinal wall. In contrast no metastasis was observed in mice implanted with MUC4 knockdown cells. In an independent experiment, larger tumors were obtained by orthotopically implanting 3× more MUC4 knockdown cells (0.3 × 10^6^). However, these tumors, while comparable in size to the previously obtained control tumors (0.75 g), were still incapable of distant metastasis, suggesting that the differences in the metastatic potential of control and MUC4 knockdown cells is independent of the size of the primary tumor (Table S1).

### Triple Negative Breast Cancer Tissues Overexpress MUC4 Mucin

**Figure 6:**
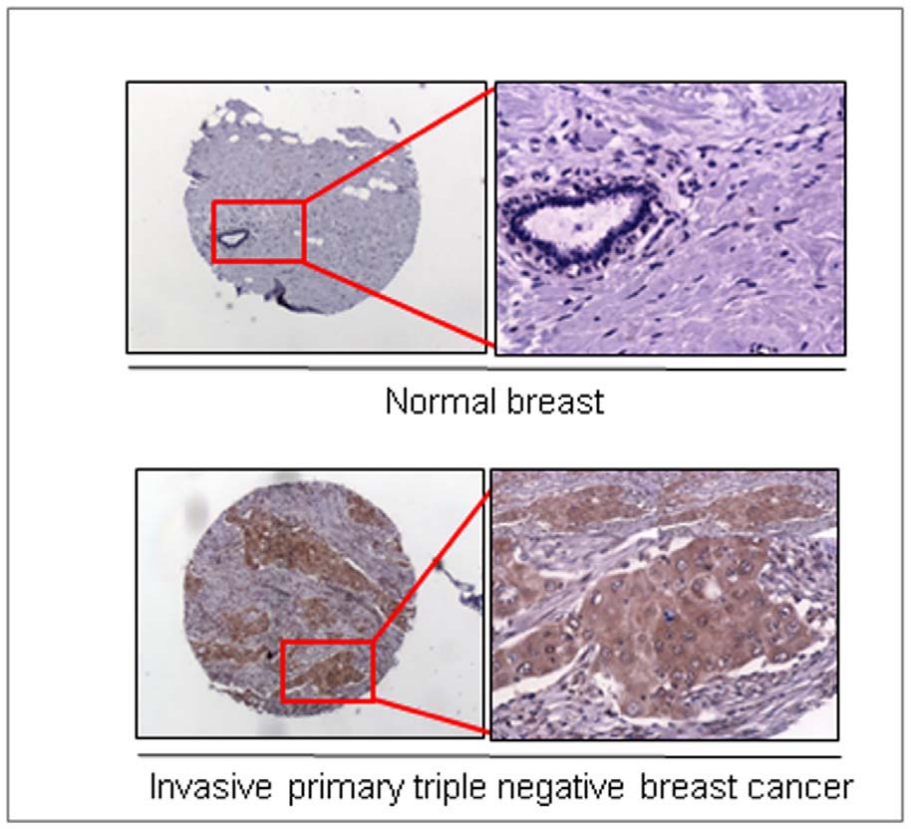
Differential over-expression of MUC4 mucin in TNBC tissues compared with normal breast tissues. Immunohistological analyses were performed using the anti-MUC4 mouse monoclonal antibody (2214, generated in our laboratory, against a sequence close to the N-terminus of human MUC4) on tumor microarrays (BR1503 and BR10010) containing normal breast and invasive TNBC tissues and observed under a Nikon light microscope. MUC4 expression in invasive primary (n = 35) TNBC tissues were compared with normal breast tissue (n = 6) in a set of arrays. High immune-reactivity for MUC4 was detected in invasive TNBC tissues, but not in normal breast tissues. The image presented was taken at 4× magnification, and the higher magnification images (marked with a red box) were taken at 10× magnification.

To determine the clinical relevance of MUC4 expression in TNBCs, immunohistological analyses were performed on TNBC tumor microarrays using anti-MUC4 antibody [37]. While expression of MUC4 was not detected in normal breast tissues, primary invasive TNBC tissues were found to be positive for MUC4 expression (**Figure** 6). A total of 35 primary tissues, and 6 normal breast tissues were examined. No expression of MUC4 was observed in the normal breast tissues (0/6, composite score 0±0), however, 54% (20/35, composite score 2.7±1.3, p = 0.018) primary invasive TNBC tissues were found to be positive for MUC4 expression. Composite score calculated based on only 20 MUC4 positive samples was 4.8±2.7, p = 0.0002.

### MUC4-associated Gene Expression, Pathways, and Interaction Networks

**Figure 7:**
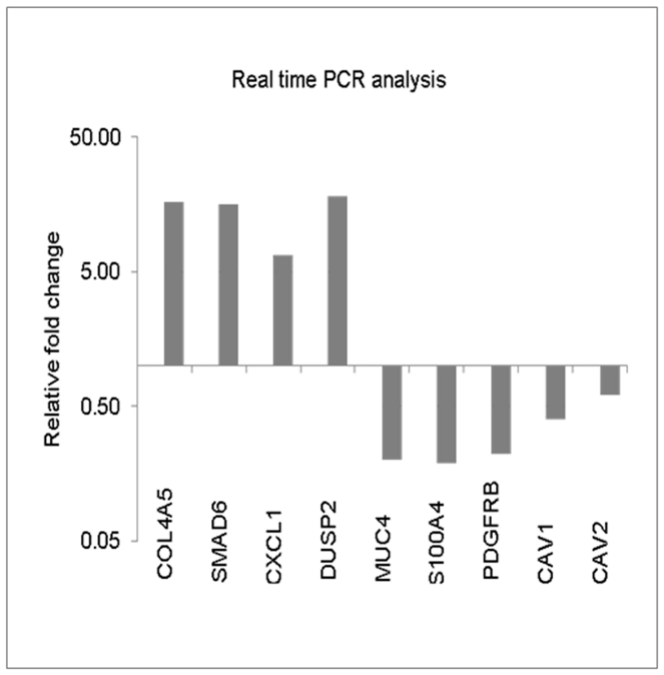
MUC4-associated gene expression, pathways, and interaction networks in MDA-MB-231 cells. Array data were validated using RT-PCR using specific primers: 20 ng mRNA from control and MUC4 knockdown cells were reverse transcribed and used for RT-PCR using MUC4 specific primers and the LightCycler SYBR Green 1 Master. The β-actin specific primers were used as control. CT values were calculated and plotted to verify the up-regulated and down-regulated genes.

We investigated alterations at transcript level following MUC4 knockdown in MDA-MB-231 cells using human genome microarray analysis. A total of 175 genes exhibited a >2 fold differential expression in MUC4 knockdown cells compared with control cells. The top-scoring network of interactions among the differentially expressed genes in control versus MUC4 knockdown cells is shown along with the table that lists statistically significant enriched high-level functions (**Figure** S3). In agreement with the results presented in **Figure** 2C, the Erk1/2 and MAPK nodes were highly perturbed upon MUC4 silencing (**Figure** S3). Selected genes that exhibited the most differential expression in MUC4 knockdown cells are listed in **Figure** S4A-C. Analyses of the data revealed that several genes associated with cellular motility, proliferation, inflammatory response, and cellular signaling, were differentially regulated in MUC4 knockdown cells. Some important genes *COL4A5*, *SMAD6*, *CXCL1*, and *DUSP2* were found to be up-regulated and several other genes *A100A4*, *PDGFRB*, *CAV1*, and *CAV2* were found to be down-regulated and hence were validated (**Figure** 7). The results of the real time analyses were in complete agreement with the microarray data, indicating that these genes could be involved in mediating the modulation of signaling pathways by MUC4.

## Discussion

Although there is a high incidence of MUC4 expression in breast cancer [15] and a significant association with metastatic disease [16], limited information is available regarding its functional role(s) in breast cancer especially in the triple negative sub-type. We have demonstrated previously that the MUC4 promotes cell proliferation and survival by binding to the receptor tyrosine kinase ErbB2 and activating downstream signaling [10], [25]. This present study represents the first effort to define the functional roles of human MUC4, specifically in invasive TNBC cells.

Knockdown of MUC4 in MDA-MB-231 cells resulted in a reduced growth rate, indicating that MUC4 augments cell proliferation. Previous studies from our laboratory have indicated that MUC4 augments proliferation and motility of pancreatic and ovarian cancer cells [7], [10], [12], [38]. In cancer cells, proliferation is mostly driven by altered cell cycle progression [32]. Increased accumulation of MUC4 knockdown cells in the G0-G1 phase suggests that MUC4-dependent signaling mediates cell cycle progression in MDA-MB-231 cells. Thus, we have shown that MUC4 augments proliferation by regulating cell cycle progression without altering apoptosis. Further investigation revealed that enhanced cell cycle progression is partly mediated by sustained expression of β-catenin, and thereby increased expression of cyclin D1 in control cells. MUC4 augmentation of cell cycle progression is also supported by our earlier findings in pancreatic cancer cells [7].

Among four members of the ErbB family, EGFR and ErbB2 play major roles in different types of breast cancer [39]–[43]. The majority of TNBCs over-express EGFR [44], [45], and are therefore candidates for anti-EGFR therapies [46]. Our earlier studies demonstrated that MUC4 regulates ErbB2 expression by enhancing its stability without affecting its transcription or mRNA stability [25]. Unlike EGFR, ErbB2 is present at low levels in TNBC cells. Here, for the first time, we showed that MUC4 regulates the expression of all EGFR family receptors excluding ErbB4. This result suggests that MUC4 is using an alternative mechanism to promote the aggressiveness of TNBC cells. However, the effect of MUC4 on the levels of other receptor tyrosine kinases needs further research. Sprouty 2 attenuates EGFR ubiquitination and endocytosis, and therefore enhances Ras/ERK signaling [47]. We observed that MUC4 mucin increased Sprouty 2 expression, and thereby potentially prevents the ubiquitin-mediated degradation of EGFR. The increased level of EGFR in MDA-MB-231 cells potentiates growth promoting downstream signaling cascades, as evident from phosphorylation of ERK1/2 and up-regulation of PKC-γ. Further evidence of the enhanced oncogenic potential of MUC4 expressing cells was shown by their ability to undergo anchorage-dependent and -independent growth, a trait commonly used to determine the oncogenicity of cells *in vitro* [48]. Obvious increases in soft agar large colony formation were observed in control compared with MUC4 knockdown cells. Large colony formation in soft agar was inhibited by MUC4 knockdown, indicative of MUC4 dependence. Overall, analyses of soft agar colony formation together with functional indicators of oncogenesis show that MUC4 promotes oncogenic phenotypes of MDA-MB-231 cells.

Motility and invasion are also major events in the metastasis of cancer [49], [50], and are associated with poor prognosis in patients with cancer. Additionally, the migratory and invasive potential of cells are typically associated with the reorganization of actin and thereby formation of lamellipodia [51]. However, the mechanisms associated with cell invasiveness remain poorly understood. Here, we demonstrate that MUC4 promotes the migratory and invasive potential of TNBC cells (**Figure** 3). Knockdown of MUC4 in MDA-MB-231 cells reduced migratory and invasive behaviors *in vitro* by cytoskeletal rearrangement, specifically by suppressing F-actin formation. Noticeably, MUC4 knockdown cells exhibited smaller pseudopodial projections, while control cells showed long pseudopodial projections. The rearrangement of F-actin is associated with EMT and, therefore, increases motility [52]. Here, we showed decreased F-actin formation along with reduced motility in MUC4 knockdown cells. Furthermore, elevated expression of focal adhesions kinase (FAK) in human breast tumors has been correlated with increased malignancy and invasiveness [53]. MUC4 knockdown cells also had reduced phosphorylation of FAK, which possibly contributed to reduced motility of these cells. β-catenin, through its re-localization from membrane cadherin complexes to the nucleus, can act as a co-transcription factor and signaling molecule, and induces epithelial cell migration [54]. We observed decreased β-catenin expression in MUC4 knockdown cells, which further explains reduced motility and EMT in these cells. EMT promotes dissemination of a single carcinoma cell from the sites of primary tumors to distant organs (metastasis). In addition, EMT is required for normal mammary gland development [55] and plays a major role in breast cancer progression [56]. We observed that MUC4 knockdown resulted in increased expression of epithelial markers and decreased levels of mesenchymal markers, suggesting that MUC4 knockdown switches MDA-MB-231 cells to an epithelial from a mesenchymal phenotype. This observation is in agreement with our recent findings that showed the involvement of MUC4 in EMT in ovarian cancer cells [57]. Analyses of oncogenic behavior using biologically relevant and sensitive 3D Matrigel cultures showed that MUC4 expression in MDA-MB-231 contributes to their oncogenic potential. Although MUC4 knockdown did not induce structural polarization, organized structures reminiscent of mammary gland acini did appear. The process of metastasis is a complex phenomenon regulated by many components that facilitate the detachment of tumor cells from primary tumors to secondary sites [49]. Our results indicate that MUC4 may potentiate the metastatic behavior of TNBC cells by inducing cytoskeletal rearrangement. The decreased proliferation rate, motility, and invasive potential observed *in vitro* following MUC4 knockdown resulted in reduced tumorigenicity and metastatic potential *in vivo*, when control and MUC4 knockdown cells were orthotopically implanted in mice. This strongly indicates that MUC4 is associated with the oncogenicity of MDA-MB-231 cells. Our experimental findings are in agreement with the expression profile of MUC4 in a small set of clinical TNBC samples. We observed that MUC4 is over-expressed in invasive TNBC tissues, but not expressed in normal breast tissues. These preliminary studies provide a strong rationale to undertake a future study in a larger sample set to determine the association of MUC4 with TNBC and other breast cancer sub-types. Taken together these findings suggest an important role for MUC4 in tumorigenesis and metastasis of TNBCs.

To further understand MUC4-mediated oncogenic signaling pathways, we studied global alterations in gene expression. Detailed analyses of microarray data further support the concept that MUC4 confers oncogenic potential to MDA-MB-231 cells. The top-scoring network of interactions (**Figure** S3) among the differentially expressed genes in control versus MUC4 knockdown cells involved Erk1/2, which is in complete agreement with the reduced levels of phosphorylated Erk1/2 observed in the immunoblotting experiments (**Figure** 2C). Moreover, we observed statistically significant changes in high-level cellular functions (**Figure** S3) including metastasis (22 genes), apoptosis (15 genes), tumorigenesis (12 genes), cell movement (17 genes), proliferation (29 genes), disassembly of focal adhesions (6 genes), and disassembly of actin filaments (7 genes). In addition, knockdown of MUC4 up-regulated membrane components such as type IV collagen (COL4A5), an antagonist of signaling by TGF-β type 1 receptor super family members (SMAD6). Knockdown of MUC4 also up-regulated DUSP2, a dual phosphatase kinase, and thereby down-regulated mitogenic signal transduction by dephosphorylating both Thr and Tyr residues on Erk1/2, and agrees well with our immunoblotting data (**Figure** 2C). Furthermore, MUC4 knockdown up-regulated CXCL1, which is a ligand of CXCR1 and CXCR2. Controlling the levels of cytokines including CXCL1 is important for controlling immune cell infiltration, and ultimately *in vivo* tumor growth [58]. The effects of increased CXCL in the current study warrant further research.

**Figure 8:**
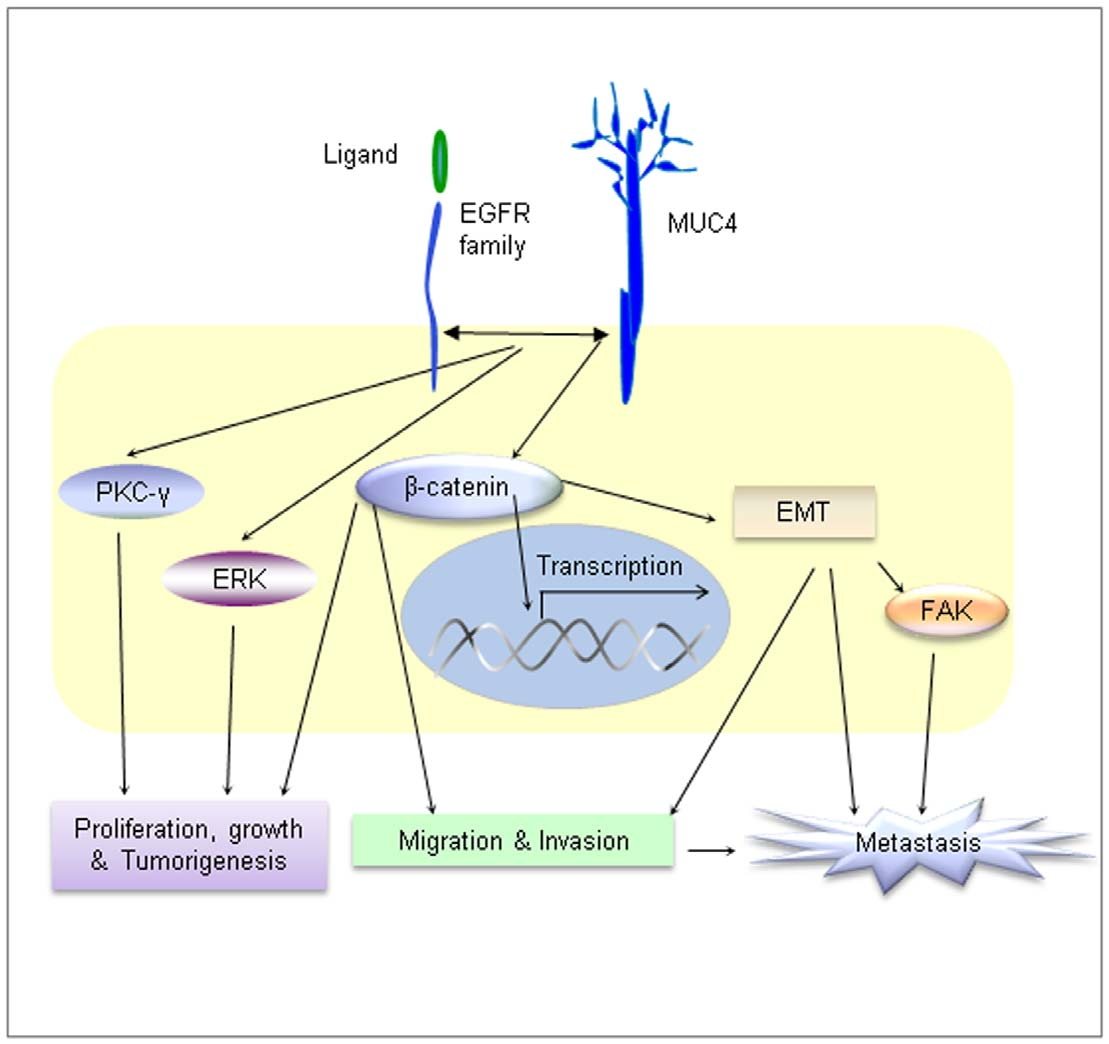
A schematic diagram showing the contribution of MUC4 in the overall aggressiveness of TNBC cells. MUC4 maintains the sustained expression of EGFR family proteins and thereby potentiates downstream signaling events mediated through the PKC-γ and Erk1/2 signaling. MUC4 maintains sustained expression of β-catenin, which induces proliferation, tumorigenesis, migration, invasion, and metastasis.

The expression of S100A4, PDGFR, CAV1, CAV2, and many other genes was down-regulated in MUC4 knockdown cells. The S100A4 protein functions in motility, invasion, and tubulin polymerization of many cell types [59]. Chromosomal abnormalities and altered expression of S100A4 have been implicated in tumor metastasis [60]. The binding of ligands (PDGF-α, -β, -γ, and –δ) activates the intracellular kinase activity of PDGFR, and initiates intracellular signaling through the MAPK, PI3-K, and PKC-γ pathways. The down-regulation of PKC-γ upon MUC4 knockdown is in agreement with our western blot data (**Figure** 2C). Caveolin expression is elevated in breast cancer and associated with both primary and metastatic breast cancer [61]. CAV1 (caveolin-1), has been identified as a marker associated with a basal-like phenotype in both hereditary and sporadic breast cancer [62], and has been proposed to play a role in intracellular cholesterol trafficking [63]. A schematic of the overall study performed indicates several pathways of involvement of MUC4 in the pathogenesis of invasive TNBCs (**Figure 8**). Altogether, these results suggest that MUC4 modulates multiple signaling pathways that confer aggressiveness to MDA-MB-231 cells.

### Conclusions

In conclusion, we have shown for the first time that MUC4 promotes TNBC cell invasive activity through EGFR family protein (EGFR, ErbB2 and ErbB3) and its downstream signaling (**Figure 8**). We have also shown that MUC4 is differentially over-expressed in primary and metastatic TNBC tissues compared with normal breast tissues. Therefore, MUC4 could be a new potential target for the treatment of invasive TNBCs.

## Supporting Information

Figure S1 **(A) Histograms of cell cycle analyses of control and MUC4 knockdown cells.** (B) Histograms of apoptosis assays of control and MUC4 knockdown cells.
(TIF)

Figure S2 **MUC4 down-regulation maintained in a tumor generated by the orthotopic implantation of MDA-MB-231-shMUC4 cells in mammary fat pads of nude mice.** (A) Real-time PCR analysis of tumor samples. A total of 20 ng mRNA from tumors was reverse transcribed and used for real-time-PCR using MUC4 specific primers and SYBR green master mix. The GAPDH specific primers were used as control. CT values were calculated and plotted. (B) Immunoblot analysis of tumor samples. A total of 50 µg of protein from tumors was immunoblotted using 8G7 anti-MUC4 monoclonal antibody.
(TIF)

Figure S3 **The top-scoring network of interactions among the differentially expressed genes in control versus MUC4 knockdown cells.** The table lists statistically significant enriched high-level cellular functions.
(TIF)

Figure S4 **Regulated mRNAs in MDA-MB-231 cells, after knockdown of MUC4, using human genome array analysis.** (A) BRB-Arraytools hierarchical clustering of genes with large fold-change. (B) Names and average log fold-change values of selected down-regulated genes. (C) Names and average log fold-change values of selected up-regulated genes. (*) real-time PCR validated genes.
(TIF)

Table S1 **Metastatic spread in nude mice when MUC4 knockdown (MDA-MB-231-shMUC4) cells were implanted (0.3×10^6^ cells) into the right 3^rd^ mammary fat pad.** No metastasis was detected in any mice injected (n = 6) with MDA-MB-231-shMUC4 cells.
(TIF)

Table S2 **List of primers that were used for real-time PCR analysis and validation of microarray data.**
(TIF)
