# Supplementary figures and images for: MUC4 Overexpression Augments Cell Migration and Metastasis through EGFR Family Proteins in Triple Negative Breast Cancer Cells
Source: PLoS One. 2013 Feb 11;8(2):e54455. doi: 10.1371/journal.pone.0054455 (PMC3569463; doi:10.1371/journal.pone.0054455)

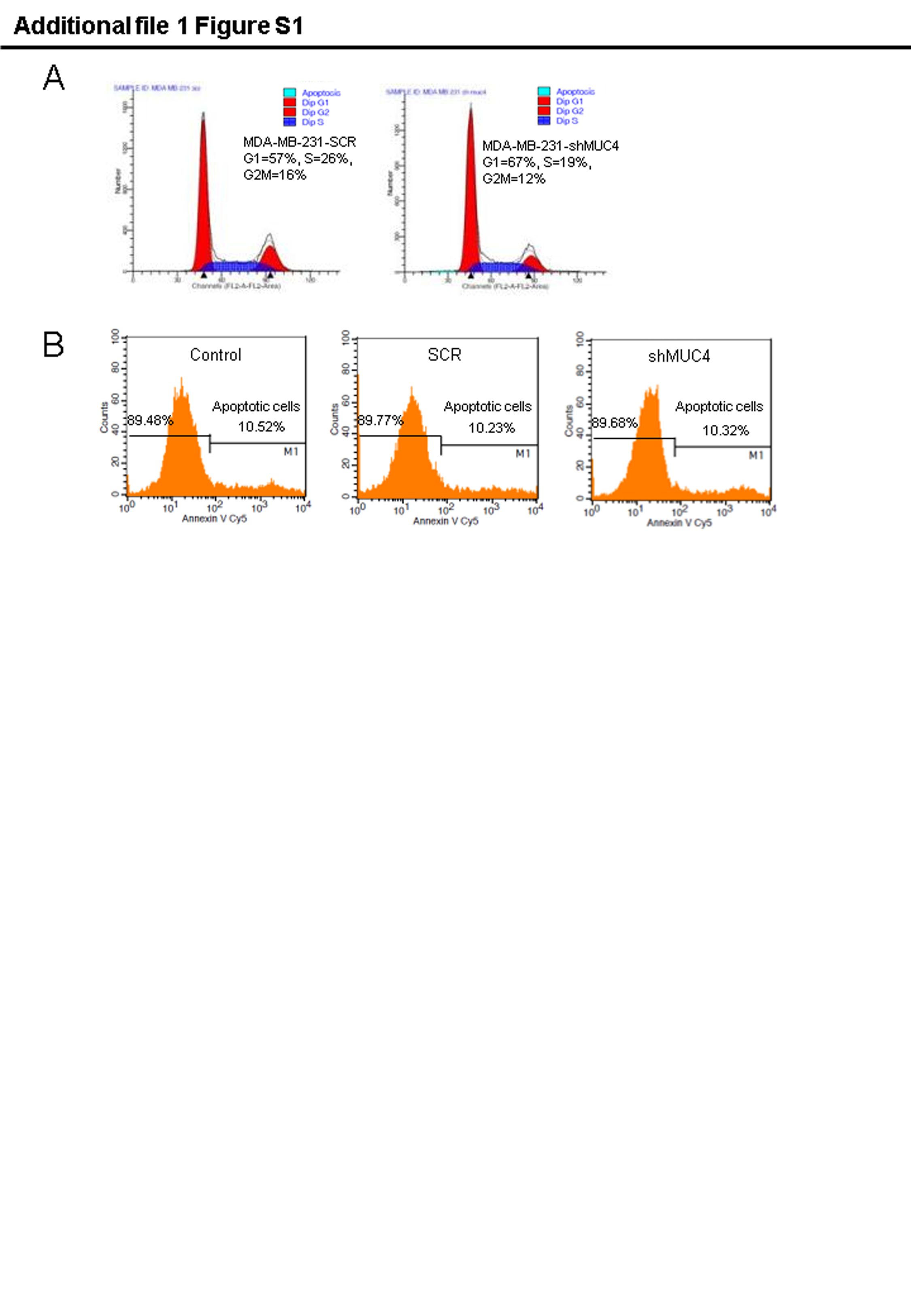

Supplement: Figure S1 — (A) Histograms of cell cycle analyses of control and MUC4 knockdown cells. (B) Histograms of apoptosis assays of control and MUC4 knockdown cells. (TIF) [file pone.0054455.s001.tif]

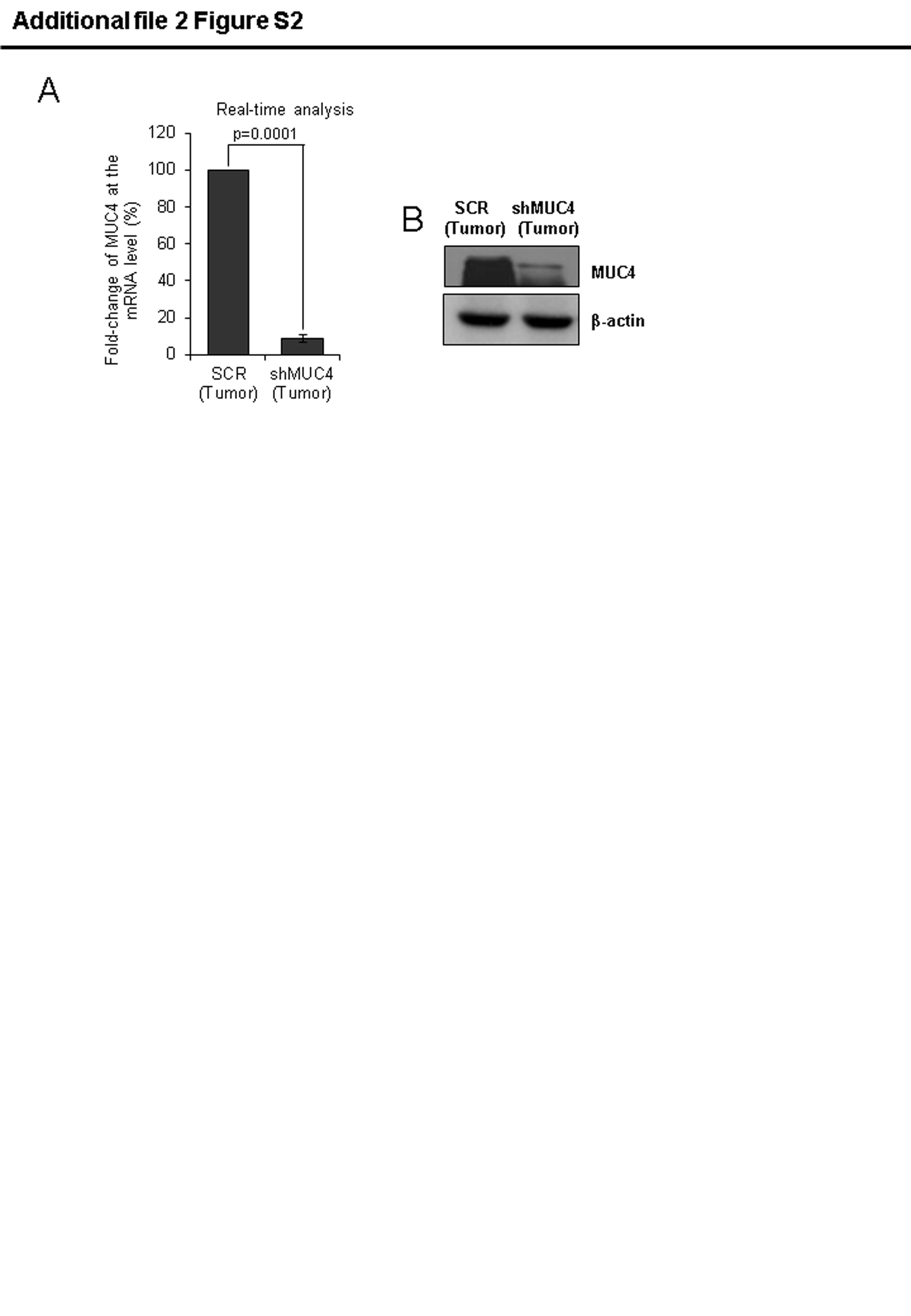

Supplement: Figure S2 — MUC4 down-regulation maintained in a tumor generated by the orthotopic implantation of MDA-MB-231-shMUC4 cells in mammary fat pads of nude mice. (A) Real-time PCR analysis of tumor samples. A total of 20 ng mRNA from tumors was reverse transcribed and used for real-time-PCR using MUC4 specific primers and SYBR green master mix. The GAPDH specific primers were used as control. CT values were calculated and plotted. (B) Immunoblot analysis of tumor samples. A total of 50 µg of protein from tumors was immunoblotted using 8G7 anti-MUC4 monoclonal antibody. (TIF) [file pone.0054455.s002.tif]

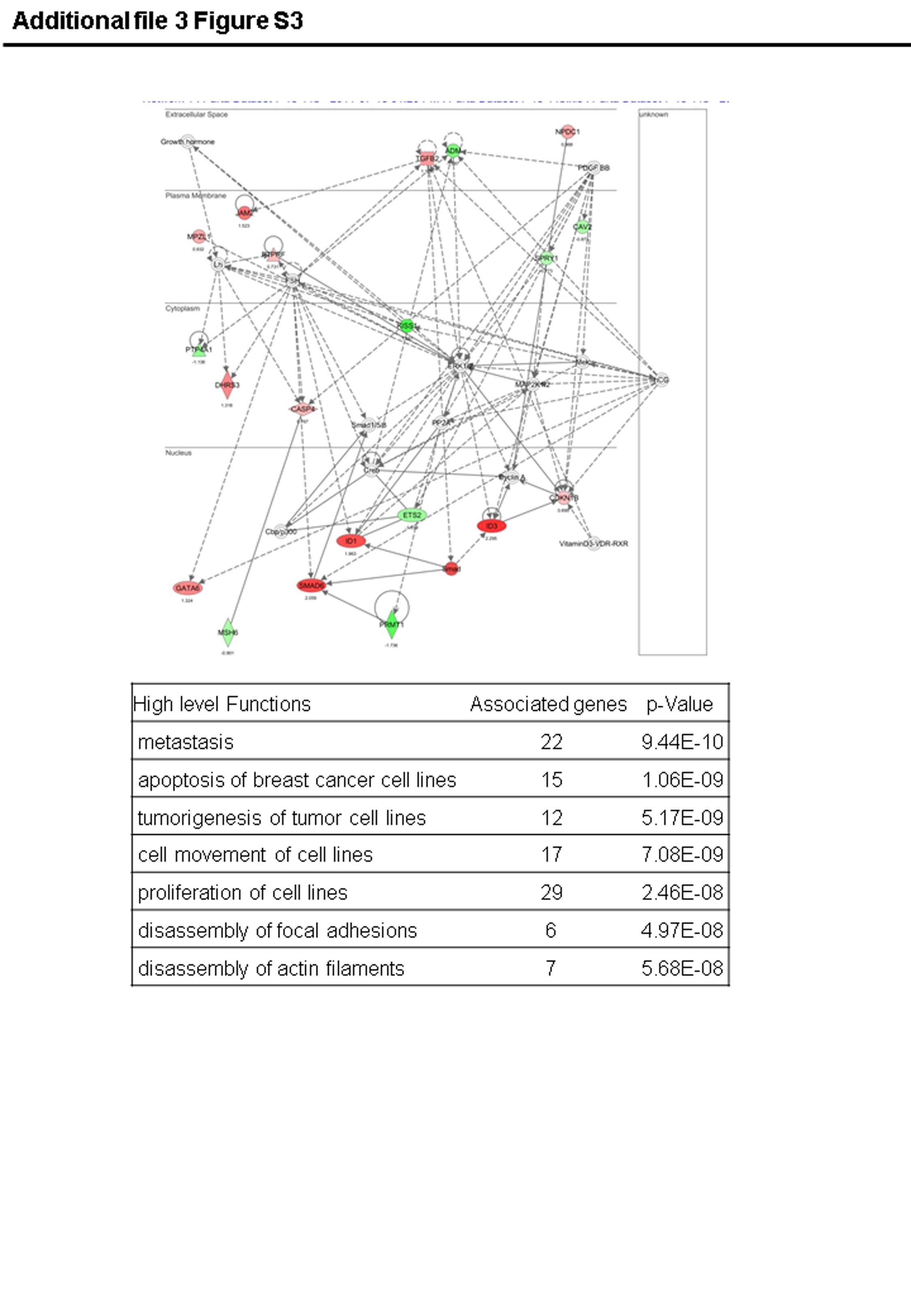

Supplement: Figure S3 — The top-scoring network of interactions among the differentially expressed genes in control versus MUC4 knockdown cells. The table lists statistically significant enriched high-level cellular functions. (TIF) [file pone.0054455.s003.tif]

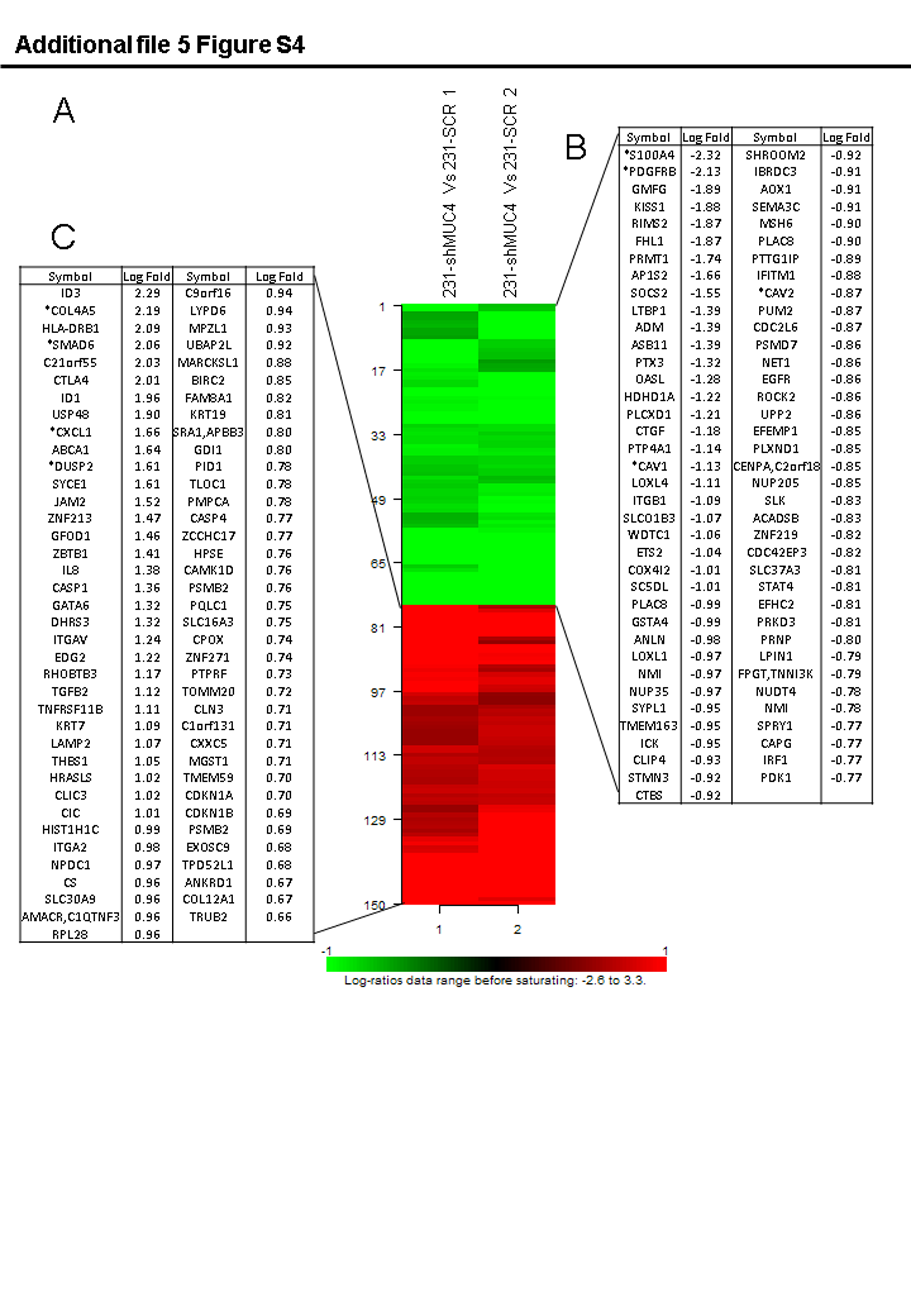

Supplement: Figure S4 — Regulated mRNAs in MDA-MB-231 cells, after knockdown of MUC4, using human genome array analysis. (A) BRB-Arraytools hierarchical clustering of genes with large fold-change. (B) Names and average log fold-change values of selected down-regulated genes. (C) Names and average log fold-change values of selected up-regulated genes. (*) real-time PCR validated genes. (TIF) [file pone.0054455.s004.tif]

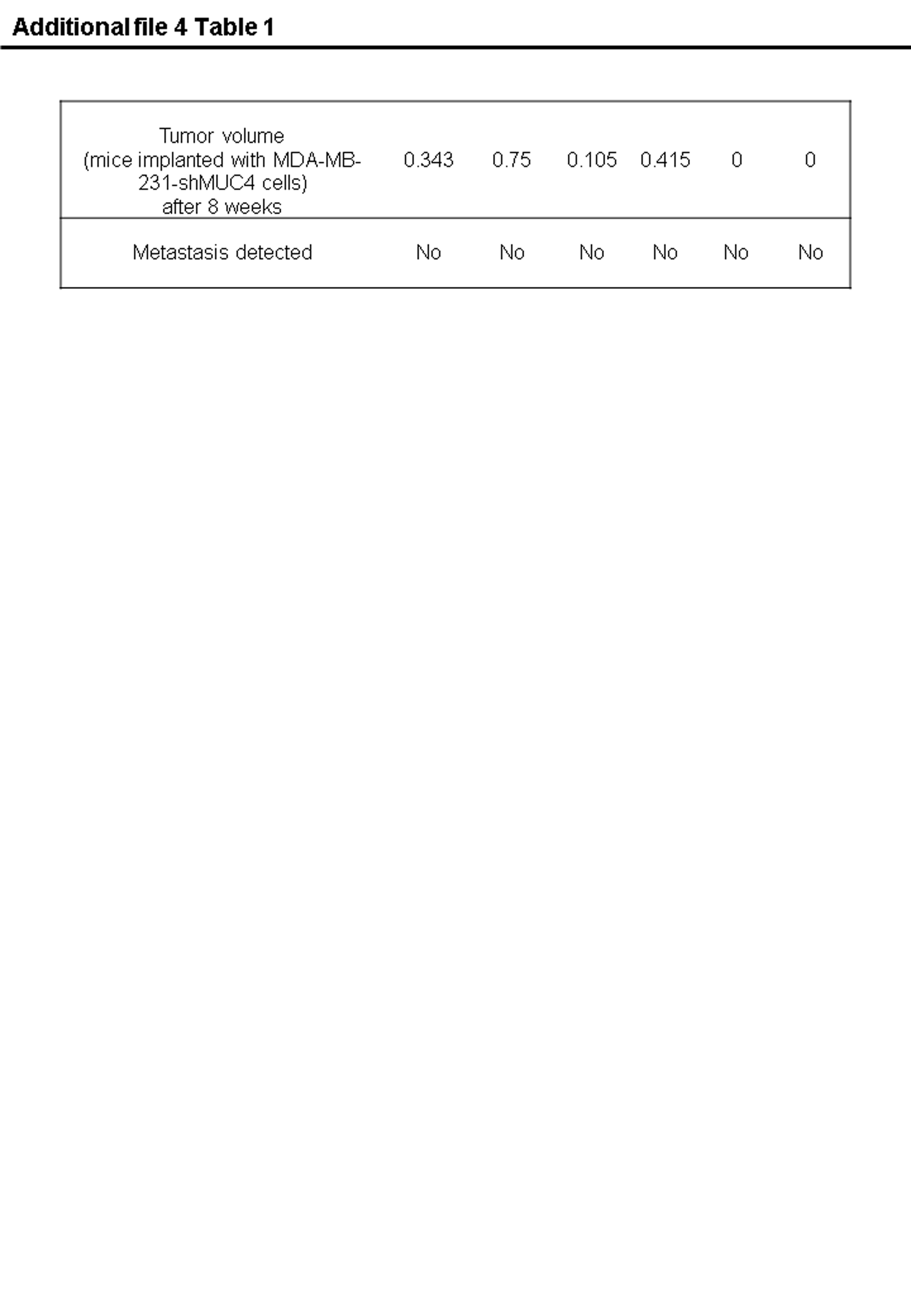

Supplement: Table S1 — Metastatic spread in nude mice when MUC4 knockdown (MDA-MB-231-shMUC4) cells were implanted (0.3×10^6^ cells) into the right 3rd mammary fat pad. No metastasis was detected in any mice injected (n = 6) with MDA-MB-231-shMUC4 cells. (TIF) [file pone.0054455.s005.tif]

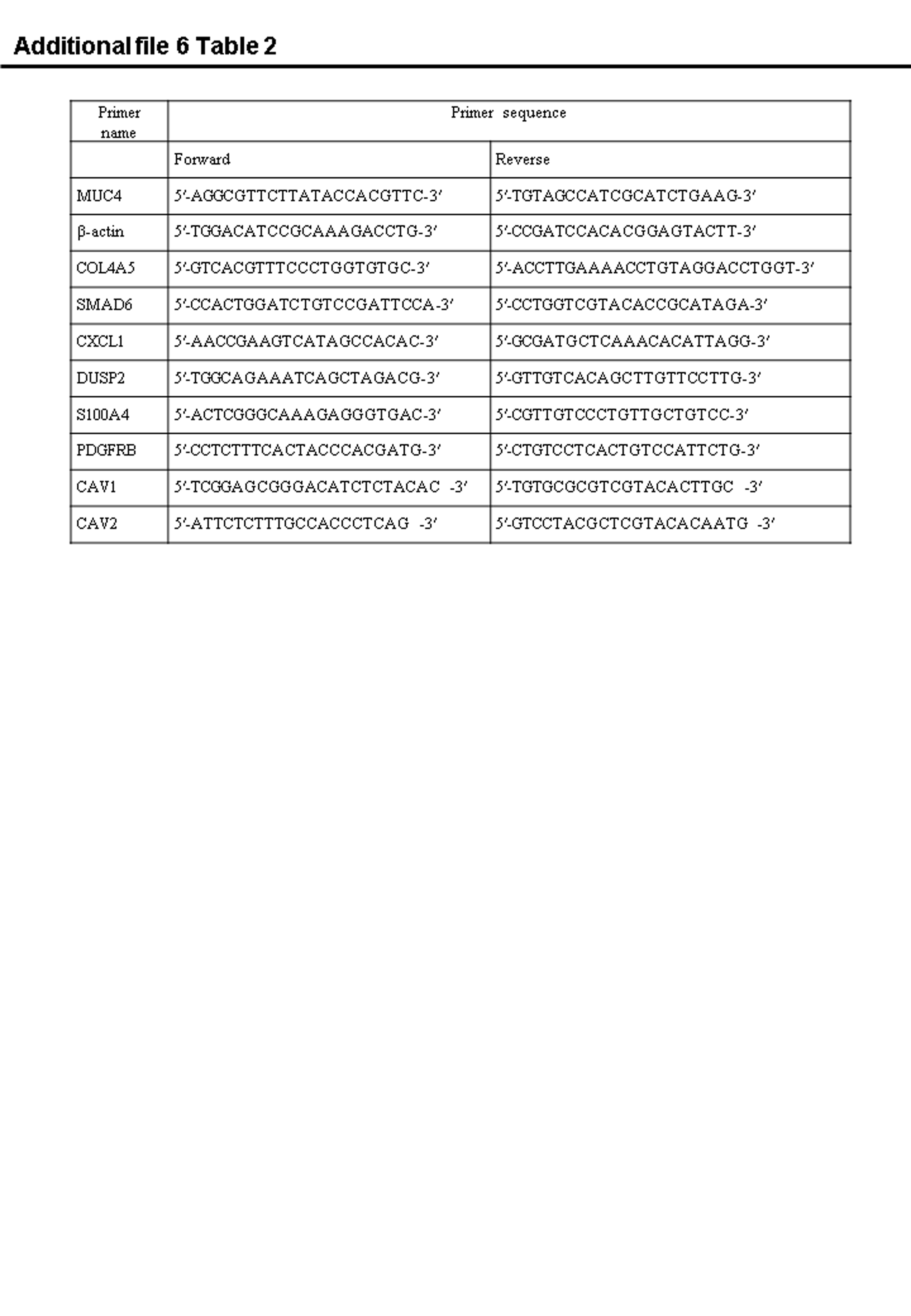

Supplement: Table S2 — List of primers that were used for real-time PCR analysis and validation of microarray data. (TIF) [file pone.0054455.s006.tif]
